# Pipeline Damage Detection Using Piezoceramic Transducers: Numerical Analyses with Experimental Validation

**DOI:** 10.3390/s18072106

**Published:** 2018-06-30

**Authors:** Shi Yan, Ying Li, Shuai Zhang, Gangbing Song, Putian Zhao

**Affiliations:** 1 Faculty of Infrastructure Engineering, Dalian University of Technology, Dalian 116024, Liaoning, China; cesyan@sjzu.edu.cn; 2 School of Civil Engineering, Shenyang Jianzhu University, Shenyang 110168, Liaoning, China; zhangshuai112358@sina.com (S.Z.); icerose1205@sina.com (P.Z.); 3 School of Architecture and Civil Engineering, Shenyang University, Shenyang 110044, Liaoning, China; 4 Department of Mechanical Engineering, University of Houston, Houston, TX 77204, USA; Gsong@uh.edu

**Keywords:** pipeline structural damage identification, piezoceramic transducers, piezoelectric elements, guided waves, pulse-echo analysis method, circumferential cracks

## Abstract

This paper aims to set up a finite element model using piezoelectric elements to realize pipeline structure damage identification analysis. Ultrasonic guided wave propagation characteristics and damage identification of pipeline structures are analyzed by the ABAQUS software. The pulse-echo method using an L(0, 2) mode impulse guided wave with a central frequency of 70 kHz is applied to evaluate different size circumferential cracks. An experiment was performed for the validation of the numerical analysis results. Both of the results show that the proposed FEM model with piezoelectric elements can efficiently reveal the dynamic behaviors, which can be used in much more precise numerical simulations than the equivalent dynamic displacement loading method.

## 1. Introduction

Pipelines are widely used for oil and gas transportation; however, pipeline leakage accidents still occur. In addition to human reasons, other factors, such as external impacts, material initial defects, environmental corrosion, internal erosion, ground surface movements, and improper maintenance, may lead to pipeline leakage. Early detection of pipeline leakage is of great importance and is becoming an important application field for structural health monitoring (SHM) technologies [1,2,3,4,5].

Piezoceramic transducer-based guided wave technology, with the advantages of a long range and accurate detection, is receiving increasing attention in pipeline SHMs [6,7]. Piezoelectric material such as Lead Zirconate Tintanate (PZT) has typical piezoelectric effects. The positive piezoelectric effect can be used to manufacture sensors, and the inverse piezoelectric effect can be applied to produce actuators. Therefore, the piezoelectric material has dual-functions simultaneously. With features of a strong piezoelectric effect, quick response, wide frequency band, low cost, and the capacity for both actuation and sensing, PZT transducers are commonly used in SHM [1,8], and PZT-based guided waves play an important role in pipeline SHM and damage detection [5,9,10].

Guided waves generated by PZT-based actuators can travel along a cylinder pipeline and will change energy and waveform as they meet damages. The PZT sensor received signal contains the damage information, and the damage location and level can be identified by effective algorithms [11,12,13,14]. Guided waves have two typical properties of frequency dispersion and multiple modes, which make the guided waves-based damage detection (DD) and SHM complicated [15,16]. An appropriate selection of the specific guided waves modes and suitable frequency range will make the damage detection and health monitoring relatively easier [17,18].

**Figure 1:**
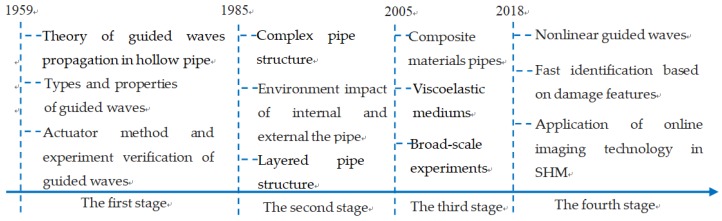
Ultrasonic guided waves damage identification stage [30,31,32,33,34].

Guided waves-based damage detection and health monitoring technologies have produced plenty of research results over a long period of development. Gazis solved harmonic guided propagation along an infinite hollow tube by use of the elastic theory, successfully explaining the dispersion effect and multi-mode phenomenon of the guided waves from the theoretical view, which marked the start of the guided waves research [19]. Greenspon systematically investigated cylinder shell dispersion curves and displacement fields [20,21]. Silk and Bainton successfully categorized the guided waves in the cylinder shell into the symmetric longitudinal mode L(0, m) and torsional mode T(0, m), and the antisymmetric flexural mode F(n, m), and the category is currently in use [22]. Lowe et al. derived the guided waves dispersion equations in a layered cylinder shell [23,24]. Aristegui et al. and Chen et al. respectively studied the guided waves propagation issues in pressured water pipelines, focusing on boundary influences on the frequency dispersion and mode transform [25,26], and Elvira further explored guided waves travelling behaviors in a circular cross-section pipe filled with viscous liquid [27]. Yan et al. experimentally validated the dispersion effect and multi-mode of guided waves [28,29]. The picture of the ultrasonic guided waves damage identification stage is seen in Figure 1.

Although finite element analysis (FEA) methods have played an important role in guided waves-based SHMs and the guided waves technology is improving, there are also some challenges with FEA on pipe structures [23,24,35,36]. One of the issues is the boundary effect on the echo signal due to inappropriate PZT transducer arrangement and frequency selections, inducing a superposition near the boundary range and making the identification very complicated. Accordingly, the main novelty was that the piezoelectric signals were not simulated by the equivalent dynamic displacement loading method but by piezoelectric elements, in which an FE model was setup using the finite element software ABAQUS to directly apply piezoelectric elements to simulate the PZT-based pipe structure damage evaluation. The method can precisely consider the impacts of piezoelectric material and connection mechanics properties on the guided wave propagation. In addition, an accurate and effective damage identification method was proposed by selecting the suitable actuation signal frequency based on the guided wave dispersion characteristics. Meanwhile, the echo signal superposition effect at pipe ends was investigated by considering the relationship between the transducer locations and wavelengths of the guided waves. To remove the superposition effect, the PZT transducer positions and the distance between PZT transducers were studied. The PZT transducers located at distances away from the end, for a certain wavelength, can effectively lower or eliminate the superposition effect. Additionally, considering the damage as a second sound source, the accurate capture of the reflected signals from the damage can improve the damage identification precision. As a consequence, the guided wave propagation process in the monitored pipeline could be more accurately simulated. To validate the efficiency of the proposed method, a model experiment was performed and the results showed the feasibility and correctness of the proposed method.

## 2. Numerical Simulation

In this section, the main work was conducted to reveal the mechanism and process of the guided wave-based pipeline SHM using FEM. Firstly, the excitation signal was chosen through analyzing the frequency dispersion curves, and described in both the time domain and frequency domain. Then, the PZT (solid elements) and pipe (shell elements) were modeled, and the distance between the PZT actuators and the PZT sensors was located. Finally, the guided wave propagation properties in the healthy pipe and the damaged pipe were investigated, respectively. These simulations were the theoretical foundation that would be verified by the experiment in Section 3.

### 2.1. Selection of Excitation Signals

**Figure 2:**
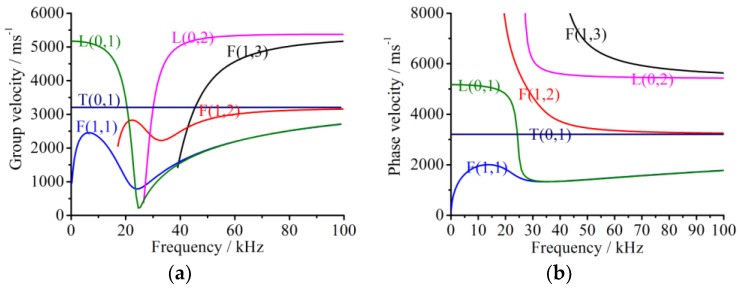
Dispersion curves of a steel pipe with an outside diameter of 70 mm and wall thickness of 4 mm. (**a**) Group velocity curves; (**b**) Phase velocity curves.

An appropriate excitation signal is an important guarantee for successful damage detection. A guided wave propagating along a hollow cylindrical shell can be divided into the longitudinal mode (L mode), torsional mode (T mode), and flexural mode (F mode), respectively. The decomposed modes of the guided waves can be controlled by a special dispersion equation which can be obtained from the Navior dynamic equations [19]. The derived dispersion equation is always a transcendental equation which can only be solved by a numerical method [22]. According to the material and dimension conditions of the pipe, the frequency dispersion curves can be obtained from a software package, named PCDISP, written in the Matlab environment, and freely available to be adapted to particular circumstances [37], as shown in Figure 2. The curves shown in Figure 2 are only the part from zero to 100 kHz for the guided waves propagating along the steel pipe with an outside diameter of 70 mm and wall thickness of 4 mm. It is obviously observed that the guided waves have typical properties of multiple modes and frequency dispersion effects.

To simplify the process of damage identification and increase the identification result precision, an L(0, 2) mode guided wave with the central frequency of 70 kHz is selected as the excitation signals. This is because (1) L mode guided waves are usually sensitive to circumferential cracks of pipes; (2) the group velocity of L(0, 2) mode has the maximum value near 70 kHz, as shown in Figure 2a, having a chance to firstly reach and be detected by sensors, which will significantly lower the identification difficulties; and (3) the selected mode in the range has a relatively weak frequency dispersion effect which is beneficial for the velocity evaluation during a damage locating process by using the time-of-flight (TOF) method. The three issues mentioned here are suggested in the paper as the rule for the guided waves selection in pipe structure damage identifications.

**Figure 3:**
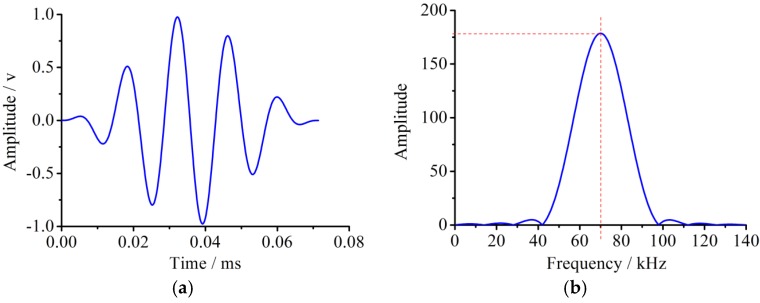
The excitation signal with the central frequency of 70 kHz and amplitude of 5 V. (**a**) The normalized waveform in the time domain; (**b**) The corresponding wave in the frequency domain.

A five-peak impulse wave has some advantages such as a narrow pulse width and less frequency dispersion. Therefore, an L(0, 2) mode five-peaks impulse wave with the center frequency of 70 kHz and the amplitude of 5 V, which is modulated by a HANNING window function, is chosen as the detection signal in this paper. The excitation signals in the time domain and frequency domain are shown in Figure 3, respectively. Figure 3a shows the normalized waveform in the time domain, and Figure 3b represents the corresponding wave in the frequency domain.

### 2.2. Establishment of Finite Element Model

During the establishment of the finite element model for a steel pipe structure, dimensions and material properties of the pipe and the PZT patches should be firstly considered. Dimension parameters of the piezoceramic patch element are shown in Table 1.

Mechanical and electrical properties of piezoceramics have a great influence on the performance of guided waves generating and propagating along the pipe structure. By bonding on the external surface of the pipe using a thin layer of epoxy, the PZT patches can form a ring shape array uniformly distributed along the circumferential direction. The thin epoxy layer was not considered for the simulation during the FEA and the PZT patches were tied together with host pipe structure element nodes at given positions. Due to piezoceramic property parameters in a small range, the parameters should be reasonably selected by the try and error method using the ABAQUS software to conduct the calculations. The selected mechanical and electrical parameters of PZT materials during the establishment of the FEA model are shown in Table 2.

The polarization directions of the selected piezoelectric element model need to be defined because of the piezoelectric material anisotropic property. In this paper, the polarization direction of the piezoelectric element model is along the thickness direction. The same direction definitions for the electrical properties are shown in Table 2.

For the tested pipe structure, its dimensions and material parameters are shown in Table 3 and Table 4, respectively.

**Figure 4:**
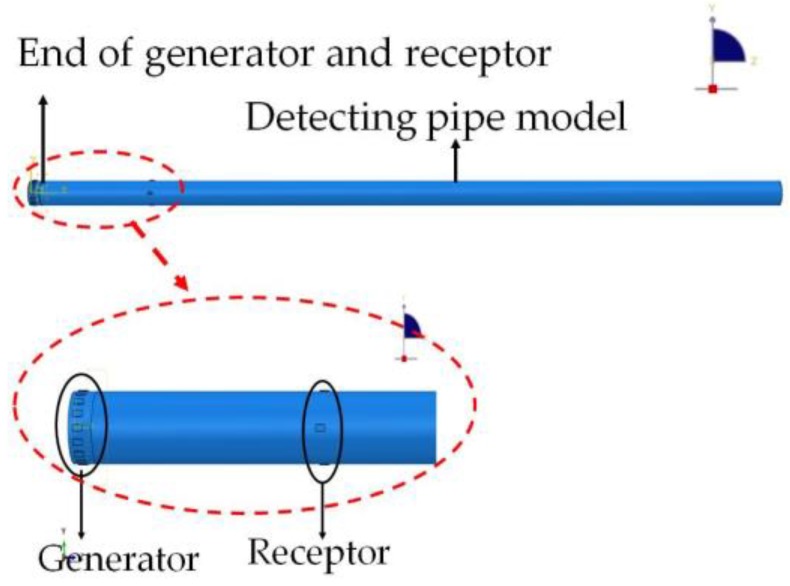
The pipe FEA model assembled with the PZT arrays.

To generate an L(0, 2) mode guided wave for identifying the circumferential crack, a group of 16 piezoceramic elements simulating the PZT patch array used as actuators are uniformly arranged along the pipe model, and another group of four piezoceramic elements used as sensors are uniformly bonded on the surface of the model at the same end. The distance between the generator array and the sensor one is 50 mm. The pipe FEA model assembled with the PZTs array and sensor is shown in Figure 4, and the layout of the PZT arrays is shown in Figure 4.

### 2.3. Selection of Analysis Setup and Time Increment

The analysis step and time increment have a great impact on the numerical results using FEM. For the issue of the guided wave-based pipe structural numerical simulation, the selection of the analysis step and time increment depends not only on the properties of the structure and damages, but also on the detection signal sampling interval and frequency. As for a larger analysis step and time increment, although the FEA calculation is time-saving, the selected detection signal might be insensitive to relative tiny damages, indicating an unexpected or even a wrong evaluation result. As for a much smaller analysis step and time increment, although it is efficient for tiny damages identification, a higher time consumption might increase the cost of applying the SHM technology in engineering. Therefore, as the dynamic implicit analysis step of the ABAQUS/Standard module is being selected to analyze the issue of pipe structure damage identification coupled with piezoceramic patches, time step and increment are selected based on prior published work and study to ensure convergence. According to the Newmark time increment scheme [38], the maximum time increment should be less than 1/20 of the period of the excitation signal corresponding to the highest frequency, as shown in Equation (1)

(1) $$\Delta t<\frac{1}{20f_{\max}},$$

where ∆*t* means the time increment and *f*_max_ represents the highest frequency of the excitation signal. In this paper, the highest frequency of the excitation signal is 70 kHz, so it can be obtained that the time increment should be less than 0.7 µm when employing Equation (1). Considering the accuracy of the analytical results, 0.1 µm is chosen as the time increment.

The output settings in ABAQUS are mainly composed of field output settings and history output settings. The simulation analysis results in the paper are selected from time history curves by extracting voltage and displacement signals in the corresponding nodes in the model.

### 2.4. Meshing

When meshing the FEM model, the C3D8E elements (an eight node hexahedral linear piezoelectric element) are used as piezoceramic elements and the S4R shell elements are applied for pipe elements. To ensure the calculation accuracy and consider the computational efficiency influence, in this paper, the grid size should be less than 1/20 of the minimum wavelength in the structure, as shown in Equation (2).

(2) $$l_{m}<\frac{\lambda_{min}}{20},$$

where *l_m_* means the size of the grid and λ*_min_* denotes the minimum wavelength of the guided wave.

Referring to Equation (2) and considering the contact type between the piezoceramic elements and the steel pipe is the “tie” type, this requires that the grid size of the slave surface should be smaller than the grid size of the main surface. Therefore, the grid size of the piezoelectric element as the main surface is set to 12 mm, and the grid size of the pipe structure as the slave surface is set to 5 mm during the meshing.

### 2.5. Simulation Result Analysis

**Figure 5:**
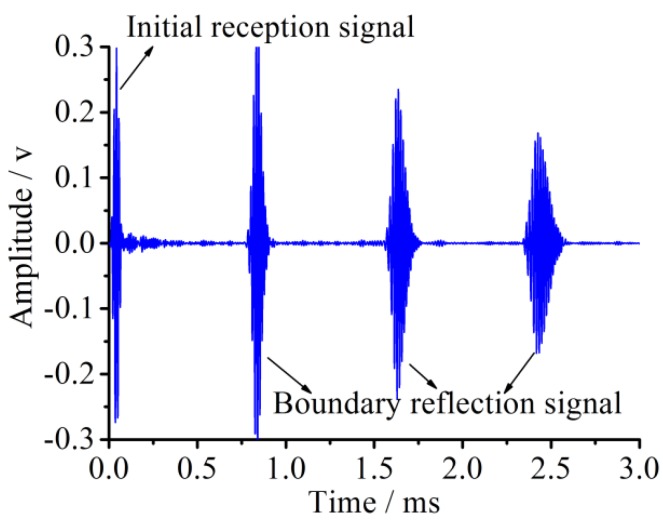
The simulation result of a received signal time history for the healthy pipe structure.

Generally, the simulations are divided into two cases according to whether damages occur or not. One case is a healthy one, which has no damages along the pipe. The other is a damaged one, where artificial damages with a certain level are placed on the given positions. The electrical analog signals for the healthy pipe structure model extracted from a piezoelectric receiving element potential are shown in Figure 5. In Figure 5, the signal packages from left to right are the received initial signal and the boundary reflection signals, respectively. It can be obtained from the result that as a signal is propagating in a healthy pipe structure, the guided wave for the same excitation central frequency will be observed for a phenomenon of decreasing amplitudes because of the energy dissipation with an increase of propagation distances and reflection times, accompanying the phenomenon of an increasing signal package length in the time domain. For each of the received signal packages, a more than five-peak impulse is clearly observed due to the boundary superposition phenomenon. This is because the PZT sensing array is so close (50 mm) to the end of the pipe structure that the later part of the sensor signal might have a superposition with the boundary reflected front part of the same sensor signal. 

**Figure 6:**
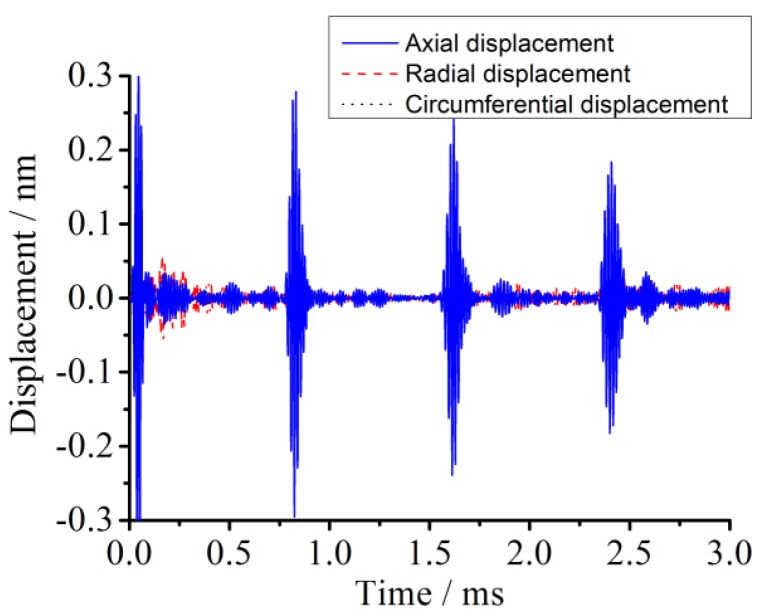
The simulation results of comparison among nodal directional displacements at the receiving signal position.

In order to verify whether the excitation signal is proximate to the expected guided wave with an L(0, 2) mode in the pipe detection simulation, three kinds of typical displacements along axial, radial, and circumferential directions at the given nodes are analyzed, respectively. Firstly, the axial, radial, and circumferential displacement curves are extracted and compared, as shown in Figure 6. Figure 6 shows that axial displacement amplitudes of the signal package at the same node in the pipe model are greater than the other two directional ones, indicating that the displacement distribution approximately complies with the L mode guided wave.

Secondly, by calculating the time difference between the initial received signal wave package and the echo signal wave one, the propagation time of the guided waves in the pipe can be calculated, and according to the position of the nodes in the pipe, the propagation group velocities of the guided waves in the pipe can be obtained, shown in Table 5.

It can be observed that the calculated group velocities values are basically the same as the theoretical value of the L(0, 2) mode guided wave at the center frequency of 70 kHz shown in Figure 2a. It is indirectly verified that the excitation signal in this simulation has approximate properties of the L(0, 2) mode guided wave.

Meanwhile, according to the calculated values of the wave group velocities shown in Table 5, the group velocities of the guided wave gradually decrease for a small certain extent (from 0.149% to 3.619%) with an increase of the signal reflection time and propagation distances, which also shows that there is an energy attenuation during guided wave propagation.

**Figure 7:**
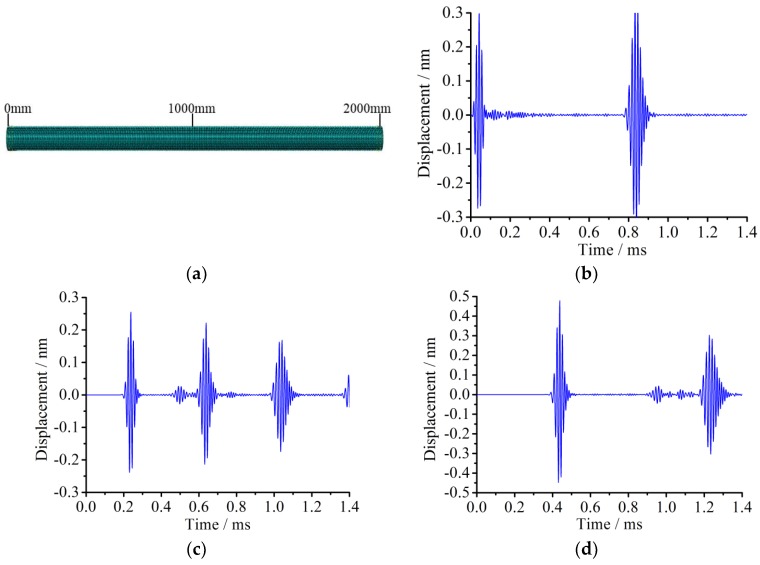
The meshing and displacement time histories at different nodes in the simulation. (**a**) The meshing of the pipe structure; (**b**) The nodal displacement time history at the position of 0 mm; (**c**) The nodal displacement time history at the position of 1000 mm; (**d**) The nodal displacement time history at the position of 2000 mm.

As the detection signal is propagating along the pipe and reflecting at the ends, the echo signal usually becomes more complicated. By analyzing the displacement time history at given nodes for the healthy pipe, it is found that the initial received signal and the echo one at the end have a phenomenon of a waveform superposition and a signal package time width broadening. It is preliminarily speculated that the superposition of the received signal occurs at areas near the ends, and signal package time width broadening comes from the frequency dispersion in the propagation process. In order to clearly explain the phenomenon, the displacement time history responses at the nodes with distances away from the excitation end of 0 mm, 1000 mm, and 2000 mm are extracted and analyzed, respectively, as shown in Figure 7.

Figure 7a shows the layout of three observation points located at 0 mm, 1000 mm, and 2000 mm from the left end. Figure 7b indicates the observed signal at the point of 0 mm. The first signal package represents the initial excitation signal and the second one is the received echo signal from the right end. Figure 7c shows the signal propagating through the point of 1000 mm. The first and third main signal packages represent the initial excitation signal and the reflection one from the right end, respectively, and the second main one represents that from the left end echo. Figure 7d denotes the signal travelling through the point of 2000 mm. The first main signal package is the initial excitation signal and the second main one is from the left end reecho. Through the displacement comparison at difference nodes shown in Figure 7, it can be concluded that the incident wave and boundary reflection ones will occur as a superposition phenomenon in the close end (connection) area.

**Figure 8:**
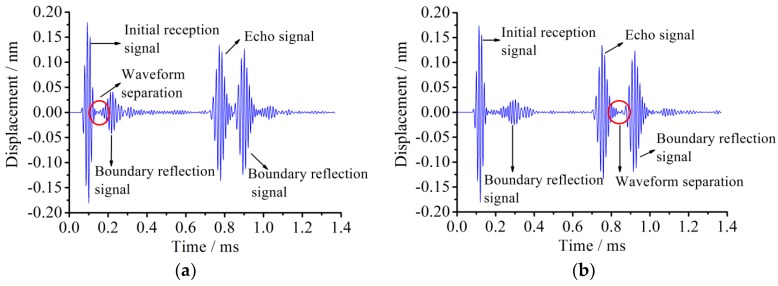
The nodal displacement signal at the beginning of separation of the incident wave and reflection wave in the simulation. (**a**) Initial reception signal; (**b**) First echo signal.

In order to determine the superposition area range, a new observation point of a signal wavelength away from the left end is selected. The node displacement of the initial received signal and its reflected signal starts clearly separating, as shown in Figure 8.

The identification of the signal separation range due to the boundary reflection superposition is important for the layout of PZT transducer arrangement. Appropriately arranging the transducer array to protect the signal from superposition may not only make installing PZT arrays much easier, but also increase the damage identification precision. In Figure 8a, the distance between observation nodes when the initial signal is separated and the connection (or end) place of the pipe is 305 mm. In Figure 8b, the similar distance for the echo signal is 415 mm. It is found that the distance for signal packages starting separation is closely associated with the signal wavelength. Equation $\lambda =c_{L}T$ shows how to calculate the wavelength of an excitation signal, where *λ* means the signal wavelength, *c_L_* represents the longitudinal wave velocity in elastic medium, and *T* denotes the period for the excitation signal.

From the above Equation, the distance between the node when the initial signal is separated and the connection (or end) of the pipe is 0.8*λ*, which means that the initial wave is prone to superposition within the range of 0.8*λ* away from the pipe connection (or end). Meanwhile, it can be also obtained that the echo wave is prone to superposition within the range of 1.1*λ* away from the pipe connection. Therefore, to avoid the superposition of the incident signal at the connection of pipe structures, the distance between the signal transducer and the connection (or end) of the pipe should be greater than the wavelength of the exciting signal.

**Figure 9:**
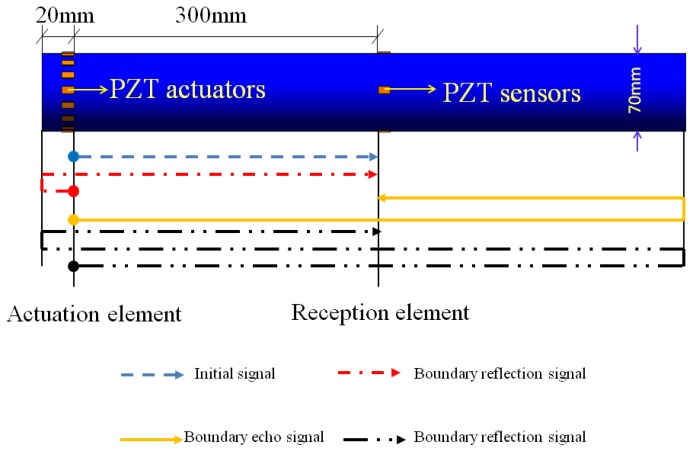
The new layout of the transducers and the schematic of signal propagation in the pipe in the simulation.

According to above analysis results, in order to avoid superposition phenomena in the connection of a pipe, excited piezoceramic elements are arranged at a distance of 20 mm from the connection of the pipe, and the received piezoceramic elements are placed at a distance of 300 mm from the connection of the pipe, and the layout of the piezoelectric elements is shown in Figure 9.

**Figure 10:**
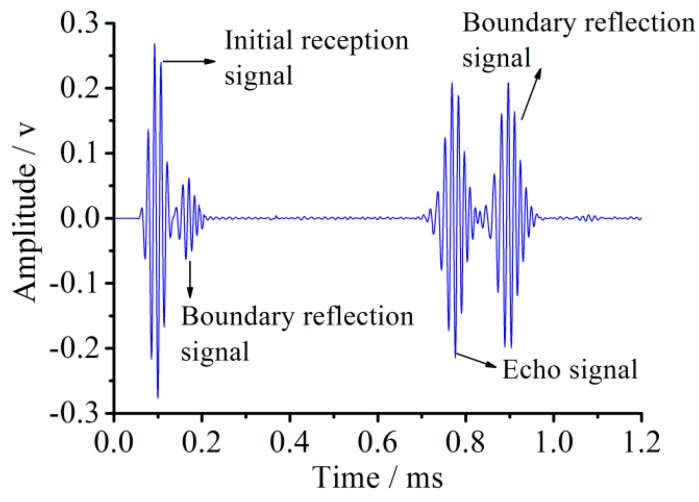
Received signal of the healthy pipe under the new piezoceramic element layout scheme in the simulation.

The received voltage signal for the healthy pipe in accordance with the new piezoelectric elements layout is shown in Figure 10.

After rearranging the excited elements and the received elements, the initial signal and its boundary reflection signal have been separated for the first and second packages from the left of the pipe, and so are the echo signals for the third and fourth packages. In this way, the difficulty of result analysis due to the superposition of signals is efficiently avoided, which will lay a foundation for the layout of the transducers and the analysis of the subsequent damaged pipes.

### 2.6. Simulation Analysis for Pipe with Circumferential Damages

Based on the above simulation research results, damage identifications of pipe structures with the damage along a circumferential direction using piezoceramic elements are carried out. The properties of the pipe model and the piezoceramic element, the model interaction, and the load boundary conditions are set according to the above analysis, as shown in Table 1, Table 2, Table 3 and Table 4, respectively.

#### 2.6.1. Circumferential Damage of Radius Angle of 45°

**Figure 11:**
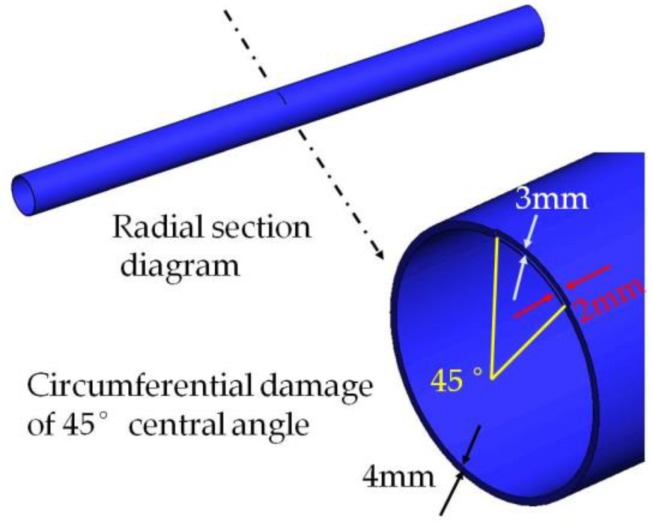
The cross-section of circumferential damage with a radius angle of 45°.

**Figure 12:**
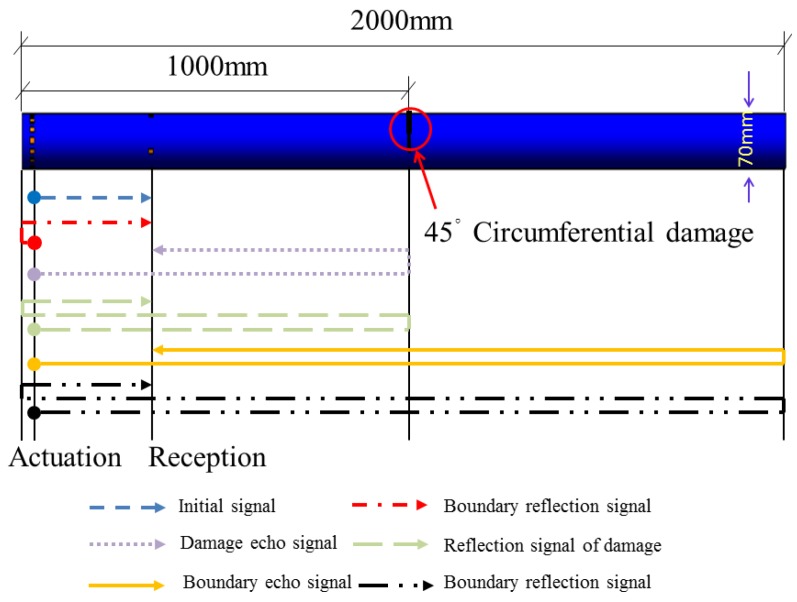
The schematic of signal propagation in the pipe with circumferential damage (45°) in the simulation.

**Figure 13:**
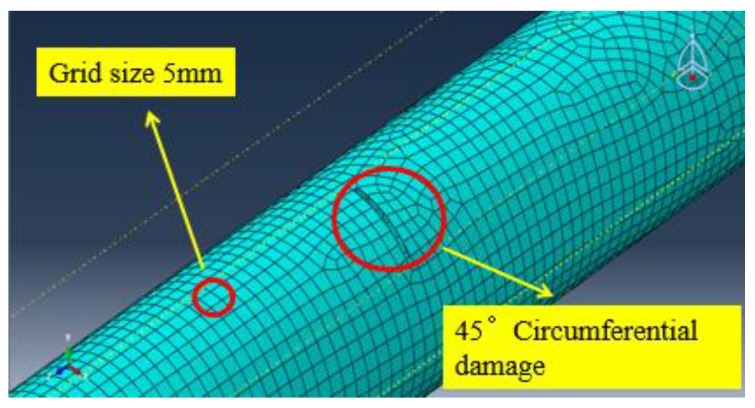
The pipe model meshing with 45° circumferential damage.

In order to investigate the propagating behavior of a PZT guided wave-based signal in a damaged pipe structure, a typical damage along the circumferential direction is introduced on the surface of the pipe. Shear type piezoceramic elements are used for the simulation of PZT transducers. A circumferential damage with the radius angle of 45°, the maximum depth of 3 mm, and the longitudinal width of 2 mm which is located at the distance of 1000 mm from the PZT actuators (700 mm from the PZT sensors) is arranged. The circumferential damage in the damaged cross-section and the location along the longitudinal direction of the pipe are shown in Figure 11 and Figure 12, respectively. Moreover, the circumferential damage is modeled in the process of “cut extrude” in the “part” model, and then the whole pipe is meshed. Thus, the nodes outside the circumferential damage were disconnected, as seen in Figure 13.

**Figure 14:**
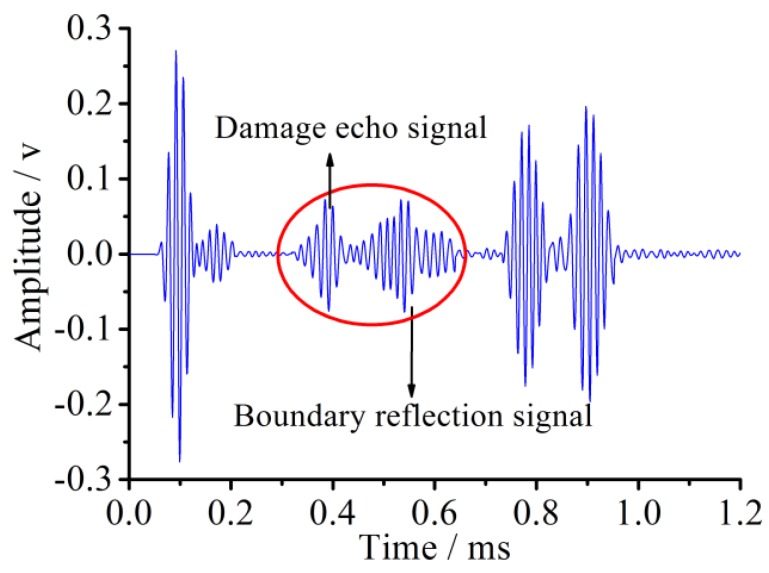
Received voltage signal in the simulation for the pipe with 45° circumferential damage.

The received voltage signal in the sensor for the pipe with 45° damage is shown in Figure 14, which reveals that two superimposed waveforms between the initial reception signal packages and the echo ones are observed when compared with the received signal of the healthy pipe. In two middle wave signal packages, the left one is the damage echo signal from the original one, and the right one is also a damage echo signal from the left boundary reflection one. The clear identification of the damage reflected signal is of great importance and efficiency because it contains useful information on the damage, such as the damage location and level, which are the two main goals of the damage identification.

Based on the principle of the pulse-echo and time of flight method, the distance between damage and the sensor can be obtained by calculating the time difference between the initial received signal and the first damage echo signal according to the group velocity of guided wave propagation along the pipe. Therefore, the evaluation value of the distance between the circumferential damage and the sensor is about 715.5 mm and the actual distance is 700 mm, and the error between the actual value and evaluation one is only 2.2%. The error might be caused by the frequency dispersion effect, which introduces a small error when evaluating the group velocity of the guided wave that is closely associated with the signal frequency.

#### 2.6.2. Change of Circumferential Damage Central Angles

**Figure 15:**
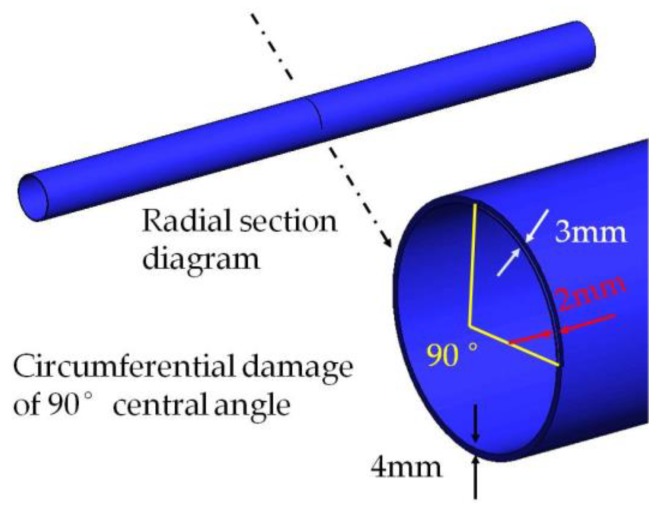
The cross-section of circumferential damage with a radius angle of 90°.

**Figure 16:**
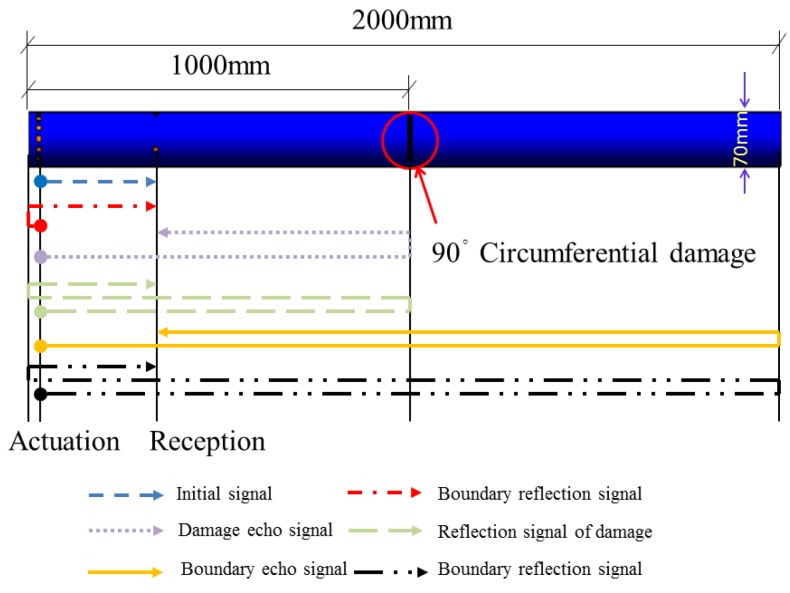
The schematic of signal propagation in the pipe with circumferential damage (90°) in the simulation.

Changing the circumferential damage center angle from 45° to 90° and keeping the other parameters unchanged, the cross section with the changed damage and the damage position along the pipe are shown in Figure 15 and Figure 16, respectively.

**Figure 17:**
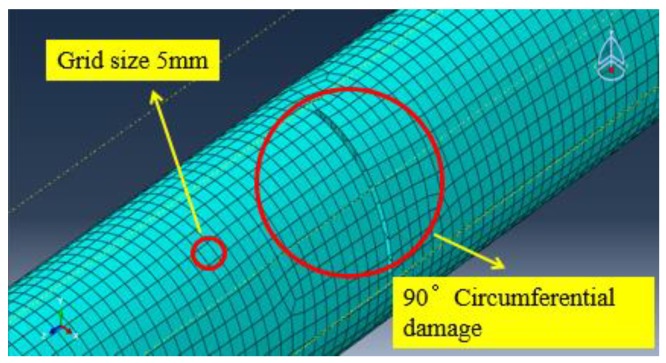
The pipe model meshing with 90° circumferential damage.

The FEA meshing of the pipe model with the circumferential damage of a radius angle of 90° is shown in Figure 17.

**Figure 18:**
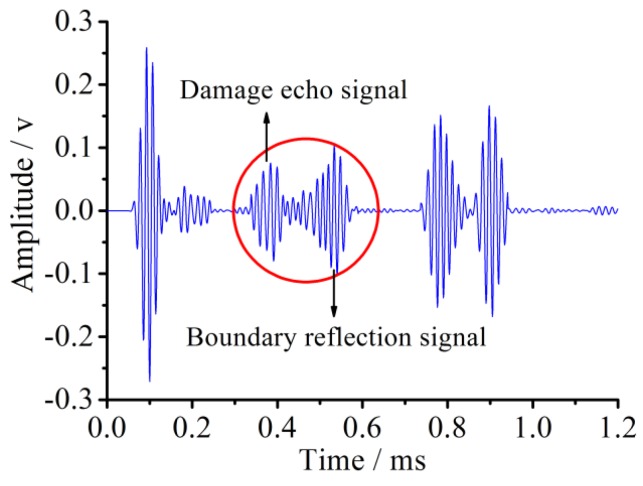
Sensor voltage signal for the pipe with 90° circumferential damage in the simulation.

The sensor voltage signal for the pipe with 90° damage is shown in Figure 18. Compared with the sensor signal of the pipe with 45° damage, the magnitude of the damage echo signal for the pipe with 90° damage is greatly increased, its time duration width is wider, and the waveform superposition effect is more serious. This is because the wider damage radius angle increases the signal reflection area and greater signal energy is reflected. The wider duration width might come from the frequency dispersion effect as the signal reflecting from the damage, and the smaller signal frequency makes the signal duration width increase a certain degree. Moreover, based on the principle of the echo method, the evaluation value of the distance between the 90° damage and the sensor is about 689 mm with the error of 1.6% compared with the theoretical distance.

#### 2.6.3. Change of the Circumferential Damage Position

**Figure 19:**
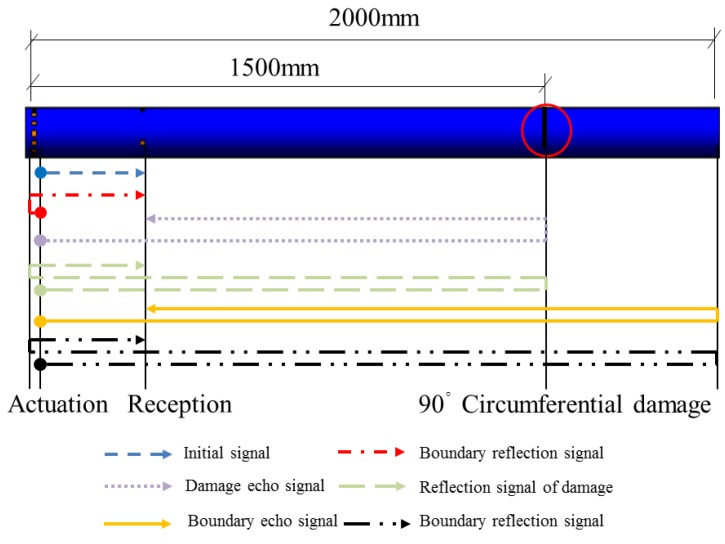
The schematic of signal propagation in the pipe with circumferential damage of 90° at the changed position in the simulation.

Removing the 90° damage position in the pipe from 1000 mm to 1500 mm away from the actuator and keeping the other parameters unchanged, the distance of 500 mm from the damage position to the right end is applied, which is close to the superposition area, and the signal superposition phenomenon is observed, which seriously affects the damage evaluation results. To receive the signal with damage information as clearly as possible, the sensors are removed to the location with a distance of 200 mm away from the excitation end (the distance between the damage and the sensor is 1300 mm), as shown in Figure 19.

**Figure 20:**
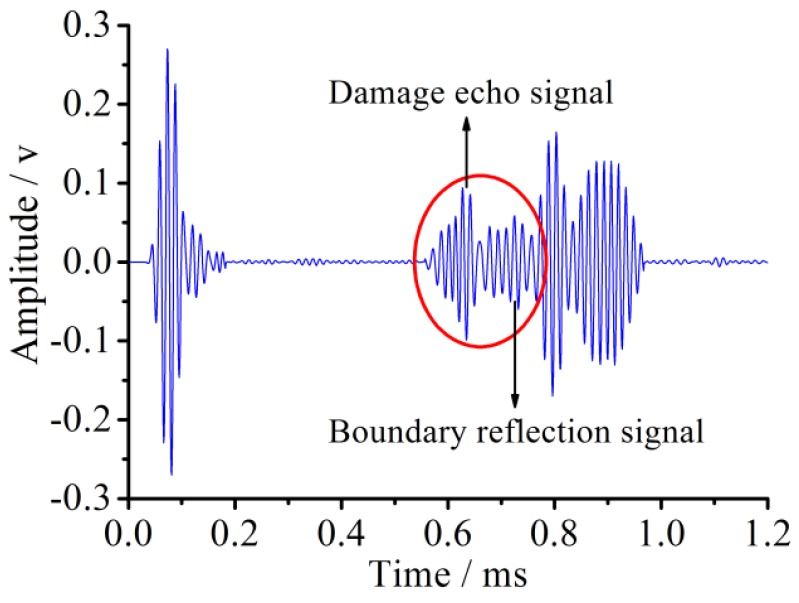
Received signal in the pipe with circumferential damage of 90° at the changed position in the simulation.

The sensor voltage signal at the new position for the pipe with the 90° circumferential damage is shown in Figure 20.

Based on the principle of the echo method, the evaluation value of the distance between the damage and the sensors is about 1325 mm with an error of only 1.9%.

## 3. Experimental Validation

### 3.1. Experimental Setup

**Figure 21:**
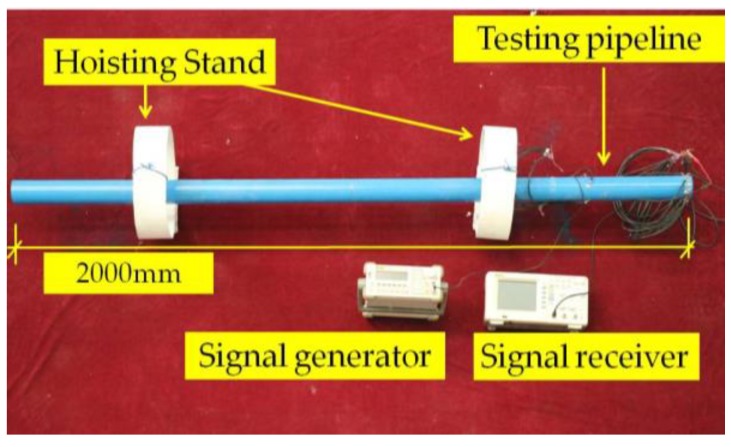
The setup of the pipe structure health monitoring test platform.

A pipe structure health monitoring test platform was established, aimed at validating the proposed FEA model and the numerical simulation results. The main experimental setup was as follows. The RIGOL DG10×2 dual channel function arbitrary waveform generator enables the output of five types of basic waveforms and arbitrary waveforms with the frequency range from 1 μHz to 5 MHz, which fully meets the requirement of the damage detection. A RIGOL DS1000 digital oscilloscope is used as the signal acquisition device in this experiment. It plays the functions of triggering, collecting, storing, and calculating the signal, as well as carrying out a 0.67 Msps sampling rate. Two groups of PZT patches (d_13_ type) and an experimental pipe were also applied. A computer was used for data processing and analysis. The setup of the pipe test is shown in Figure 21.

**Figure 22:**
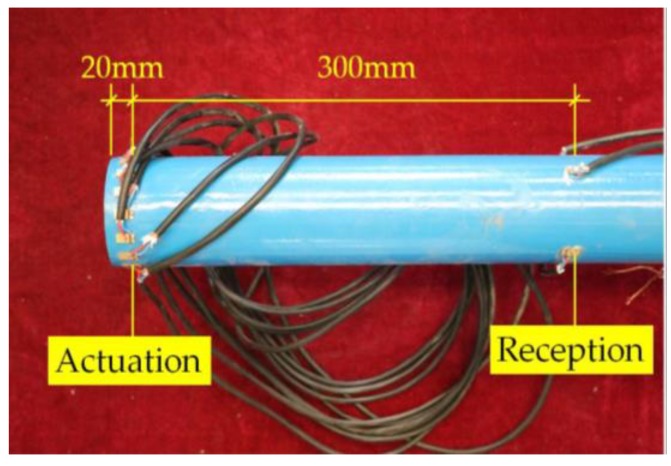
The layout of the PZT patch array on the surface of the tested pipe.

The dimension and material parameters of both the pipe and PZT transducers were consistent with the numerical model in the simulation analysis. The excitation signal in the time domain and frequency domain was shown in Figure 3. Therefore, the L(0, 2) mode five-peak wave with the center frequency of 70 kHz and the amplitude of 5 V, modulated by the HANNING window, was chosen in the test as the detection signal. The same arrangement of the PZT actuators and sensors as the simulation analysis was taken in the test. A group of 16 PZTs forming a patch array used as actuators was located on the pipe structure end to excite the mode L(0, 2). Furthermore, the 16 PZT patch array was axisymmetric in order to restrain mode T and mode F, as shown in Figure 22. The impulse wave was generated by a function arbitrary waveform generator, and then the guided wave was activated by the array of the 16 PZT actuators. After propagating in the pipe, the guided waves were received by the array of four PZT sensors. The received signal was analyzed by a computer and the useful information was obtained. The band pass filter with the cut-off frequencies of 60 kHz and 80 kHz was used during the data processing.

### 3.2. The Healthy State Pipe Experiment and Result Analysis

The experiment focused on pipe structure damage detection using the PZT-based guided wave method. According to the developed experimental system, two experimental cases of a healthy state and damaged one were considered during the experiment. The healthy pipe was used as a reference for comparison with the damaged results. The result of the healthy pipe experiment is similar to the simulation result, and the sensor voltage signal has two main wave packages before 1.5 ms. The first package represents the initial reception signal and the second denotes the boundary reflection signal. Compared with the initial reception signal, the boundary reflection signal has a smaller amplitude due to the signal energy attenuation and wider time duration because of the frequency dispersion effect.

But the energy attenuation in the experiment is a little higher than in the simulation. The signal superposition and the signal time duration broadening phenomena for the experimentally received signal are more obvious because of the more complex environment for the experiment than for the numerical simulation.

### 3.3. The Circumferential Damage Pipe Test and Result Analysis

#### 3.3.1. Test for Pipe Structure with 45° Circumferential Damage

**Figure 23:**
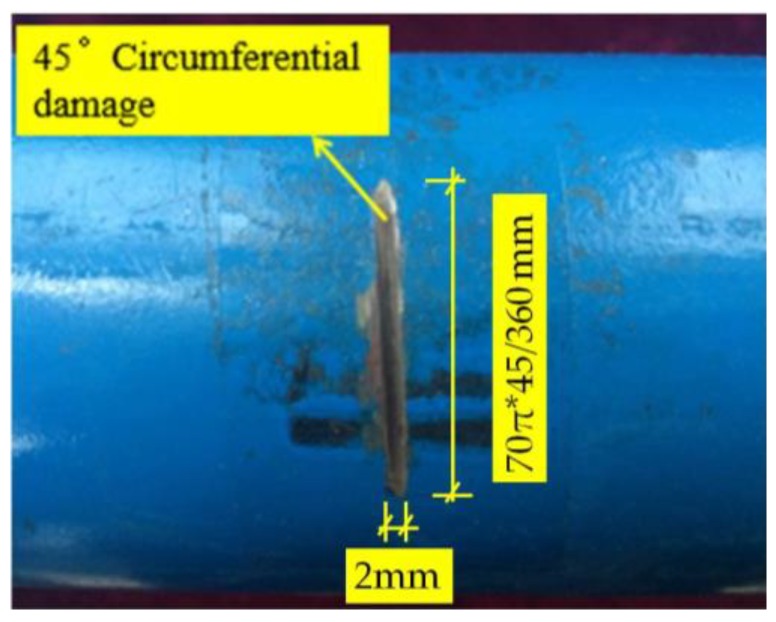
Artificial circumferential damage with the radius angle of 45° in detail.

Artificial circumferential damage with the dimension of 2 mm × 3 mm (width × depth) and the radius angle of 45° was introduced to the pipe structure. The distance between the damage location and the actuator was 1000 mm. The distance between the sensors and the actuators was 300 mm in Figure 22; therefore, the distance between the damage and sensors was 680 mm. The artificial circumferential damage with the radius angle of 45° is shown in Figure 23.

Meanwhile, the L(0, 2) mode five-peak wave with the center frequency of 70 kHz and the amplitude of 5 V modulated by the HANNING window function was chosen as the detection signal in the experiment in the paper. The excitation signal in the time domain and the frequency domain was shown in Figure 3. The waveform between the initial reception signal and the boundary reflection one was the damage echo signal. Based on the principle of the pulse-echo method, the calculation value of the distance between the damage and the sensors was about 980.5 mm, and the error between the actual values and calculation value was 3.2%.

#### 3.3.2. Test of Pipe Structure with 90° Circumferential Damage

**Figure 24:**
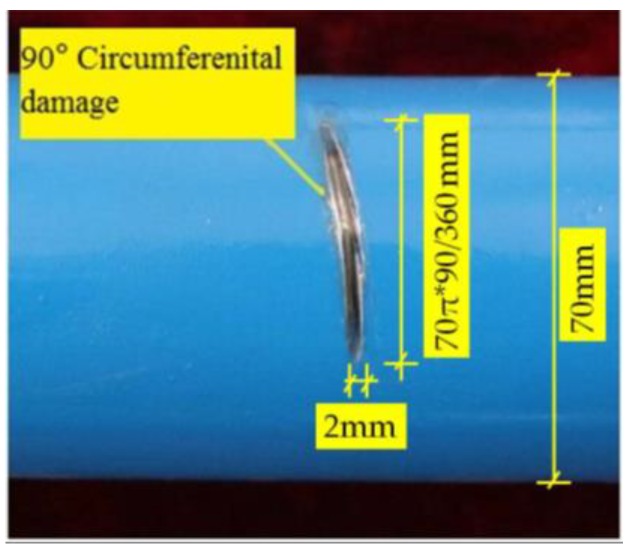
Artificial circumferential damage with a radius angle of 90° in detail.

**Figure 25:**
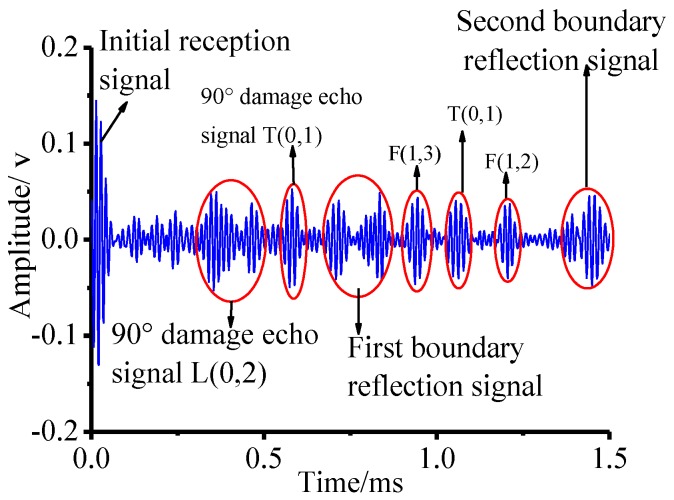
The received signal for the pipe with circumferential damage of 90° in the experiment.

**Figure 26:**
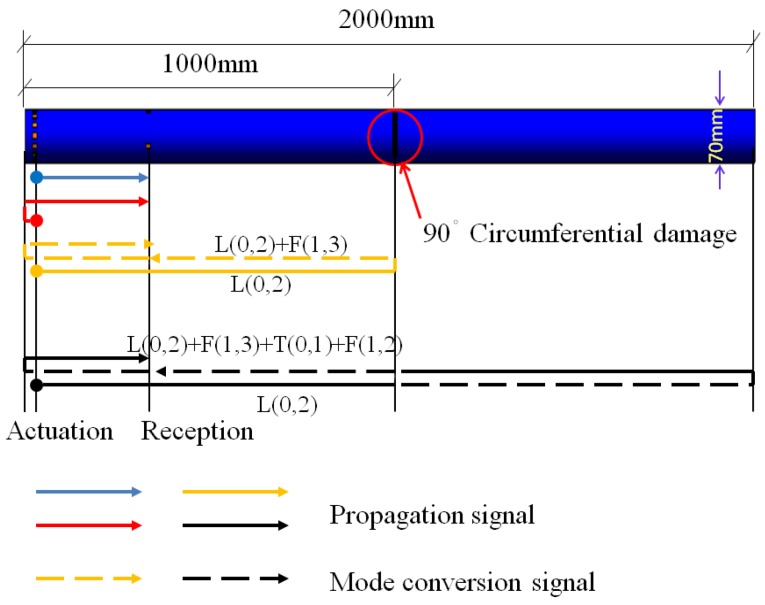
The schematic of the mode conversion signal propagating in the pipe in the experiment.

The same experimental method was used for the pipe structure with 90° circumferential damage. Changing the initial circumferential damage center angle from 45° to 90° and keeping the other parameters unchanged, the experiment was done again, and the artificial circumferential damage of 90° and the damage mean width of 2 mm are shown in detail in Figure 24. The dimension of the damage in the test was supposed to be the same as the simulation. The received signal is shown in Figure 25. In Figure 25, the waveform between the initial received signal and the first boundary reflection one is the damage echo signal. Otherwise, there was the other waveform between the damage echo signal and the first boundary reflection one. Due to the fact that the velocity of L(0, 2) mode was the fastest in the 70 kHz central frequency, there should have been no signal between the damage echo signal and the first boundary reflection. Therefore, there might be a mode conversion waveform for the actuation waveform being reflected from the damage, as shown in Figure 26. Based on the group velocity curves in the Figure 2, the mode conversion waveform might be the mode F(1, 3). Meanwhile, there were three kinds of mode conversion waveform between the first boundary reflection signal and the second boundary reflection one. The unexpected waveform of T mode and F mode might come from the actuated signal because the PZT actuators might not be absolutely symmetrically installed so that T mode and F mode could not be restrained effectively, which means a small part of the T mode or F mode signal might accompany the dominate L(0, 2). In the beginning of actuation, their amplitudes were not obvious. Their amplitudes became greater after signal propagating the damage. They were the mode F(1, 3), mode T(0, 1), and mode F(1, 2), respectively, as shown in Figure 25.

It is also found that the amplitude of the damage echo signal is obviously greater than that for the 45° damage because of the more obvious reflection effect. But the amplitude of the boundary reflection signal is smaller than that for the 45° damage, indicating that the transmission wave decreases after the increase of damage size. That amplitude depressed is analyzed using the energy method, as shown in Table 6. Based on the principle of the pulse-echo method, the calculated distance between the damage and the sensor is about 971.2 mm, and the error between the actual value and calculation one is 2.2%.

**Figure 27:**
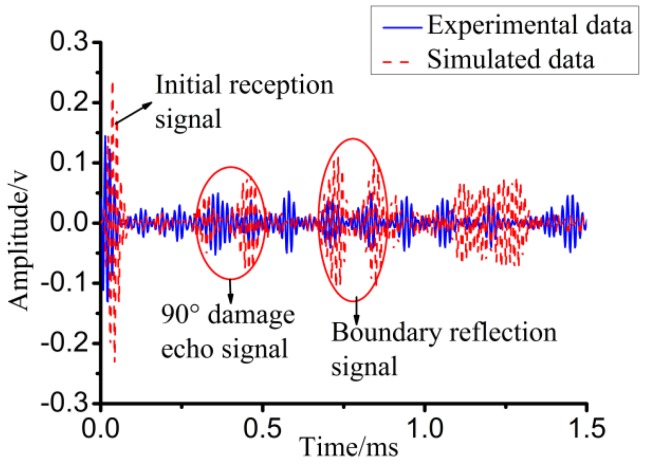
Comparison between experimental results and simulation results of pipe with 90° circumferential damage.

To verify the correctness of the pipe damage detection based on the piezoceramic element in the numerical simulation, the received experiment signal was compared with the received simulated one, as shown in Figure 27, which shows a good agreement between the experimental and the numerical results. Three signal packages represent the initial received signal, damage echo signal, and boundary reflection signal, and they match well in both amplitudes and phases. However, due to the complex environmental condition, the experimental data are obviously noisy, which makes the signal response greater than the simulation one among main signal packages. Meanwhile, the superposition effect and the time duration broadening phenomena of the received experiment signal also exist. The simulation is the most perfect condition as the PZT actuators are absolutely symmetrically and there is only one kind of mode L(0, 2) in the actuators signal. It is clear that the experimental data is more complete than simulated data.

## 4. Discussions

In general, the paper focuses on both the numerical simulation and experimental validation of the damage evaluation of pipe structures based on the piezoceramic guided wave method. Three damage cases of the healthy one, the circumferential damage with a radius angle of 45°, and the circumferential damage with radius angle of 90° were investigated. The comparison of signal package amplitudes for both the simulation and experiment is shown in Table 7. The mean value of the errors between the simulation and experiment is 0.052, which is the mean value of 0.077, 0.056, and 0.023, which can validate that the proposed FEA method is as efficient as evaluating the damage states by the PZT-based guided wave method.

To quantificationally describe the damage identification level, a signal energy-based evaluation algorithm is proposed. The entire energy of the receive signal can be expressed as the sum of the square of the value at each time step,

(3) $$E_{j}=\sum_{i=1}^{n}x_{i}^{2}=x_{1}^{2}+x_{2}^{2}+x_{3}^{2}+\cdots \cdots x_{i}^{2}+\cdots \cdots +x_{n}^{2},$$

where *x_i_* is the discretized signal data, and *E_j_* is the energy of the received signal.

To compare the difference for various signals to clearly represent the properties of the received signals, a dimensionless variable is defined as the evaluation index, shown in Equation (4)

(4) $$\alpha_{n}=\frac{E_{0}-E_{n}}{E_{0}}\times 100\%$$

where *α_n_* is the evaluation index representing the relative energy of the received signal, *n* is the number representing the corresponding load level, *E_n_* is the signal energy at the corresponding damage levels, and *E_0_* is the excitation signal energy in the healthy state.

The comparison of evaluation indexes for both the simulation and experiment is shown in Table 6. The average error between the simulation and experiment is 4.92, which validates that the proposed evaluation index can describe the damage state of pipe structures as successfully as evaluating the damage states by the PZT-based guided wave method. In order to illustrate the advantage of the evaluation index method in Table 6, the relative error was used as the indexes to compare to signal package amplitudes in Table 7. In Table 7, the relative errors of 45° and 90° damage angles were 34.35% and 13.45%, respectively. In Table 6, the relative errors of 45° and 90° damage angles were 22.3% and 11.4%, respectively. It can be seen that the method of the evaluation index shown in Equation (4) has less relative errors than the method of using signal package amplitudes as indexes.

In addition to evaluating damage levels of pipe structures, locating the damage position using the proposed method is also important. The time of flight (TOF) method is applied based on the evaluation of the signal propagation velocity and duration. The signal propagation duration is easy to identify by using experimental devices, but the key point is to evaluate which signal package among all the packages is the expected one, for a misjudgment of different signal packages might lead to a wrong evaluation of the signal propagation duration. The signal echo principle is helpful and efficient to distinguish the corresponding package properties. For the evaluation of signal propagation velocity, the guided wave mode concept can play an important role because different guided waves with different modes have various velocities which can be reflected by the guided waves frequency dispersion curves, as shown in Figure 2, as an example. For the circumferential damage identification of pipe structures, the flexural mode (L mode) and signal energy-based algorithm are strongly suggested. Although the velocity is usually supposed to be constant during the guided wave propagating along a pipe, it might be changeable because of both the frequency dispersion effect, which is defined as the velocity change with the change of signal frequency, and the mode conversion due to the boundary/damage reflection, accompanying the velocity change. The comparison of damage localization for both the simulation and experiment is shown in Table 8. The average errors for the simulation and experiment are 34.8 mm and 54.7 mm, respectively. The error may be lowered by the elaborate evaluation of the guided wave velocity by using a higher order method considering the nonlinear frequency dispersion effect.

.

## 5. Conclusions

Piezoceramic intelligent materials have been widely used in the health monitoring and damage identification of pipe structures. An SHM system for evaluating pipe structure damage levels is proposed in this paper by using ultrasonic guided wave detection technology and piezoceramic wave-based properties. The piezoelectric elements are applied for numerical finite element analysis (FEA) by using finite element software ABAQUS. To validate the efficiency of the proposed numerical model and the numerical simulation results, an experiment including several cases was performed. Some research results can be concluded, as follows.

1. An FEA model considering the couple effect of pipe structures and piezocermic patches is established and analyzed for damage identification and monitoring in which piezoelectric elements are directly used. The efficiency of the established model and the numerical results are validated by the experiment.
2. An FEA meshing method and guided wave selection rules are proposed. A grid size which should be less than 1/20 of the minimum wavelength is suggested.
3. A PZT transducer arrangement rule is put forward in which it is suggested that the PZT transducer arrays are placed at positions a length of the guided wave away from the connections (ends) to protect the received signal from superposition. The advantage of the rule is validated by both numerical and experimental results.
4. The L(0, 2) mode guided wave is suggested to be used in pipe structure damage identification with circumferential cracks. The detection signal frequency can be selected based on the corresponding group velocity dispersion curves in which the part with the maximum group velocity and weak frequency dispersion are suggested. In the paper, the guided wave with the central frequency of 70 kHz is used as an example.
5. A PZT guided wave-based experiment system for pipe structure damage identifications is proposed and validated. The damage in the pipe structure can cause or aggravate mode conversion of the echo signal.
6. A signal energy-based identification algorithm is proposed. The algorithm is successfully validated by both the numerical simulation and experiment results.

## Figures and Tables

**Table 1:** Dimension parameters of piezoceramic elements.

	Length (mm)	Width (mm)	Thickness (mm)
Piezoceramic elements	12	6	1

**Table 2:** The mechanical and electrical parameters of PZT materials ^1^.

Dielectric Constants (F/m)	Elastic Constants (N/m^2^)	Piezoelectric Constants (C/m^2^)	Density (kg/m^3^)
D11 8.11 × 10^−9^	D1111 1.21 × 10^11^	e2 23 12.3	7600
D22 8.11 × 10^−9^	D1122 7.54 × 10^10^	e3 11 −5.4	
D33 7.35 × 10^−9^	D2222 1.21 × 10^11^	e1 13 12.3	
	D1133 7.52 × 10^10^	e3 22 −5.4	
	D2233 7.52 × 10^10^	e3 33 15.8	
	D3333 1.11 × 10^11^		
	D1212 2.26 × 10^10^		
	D1313 2.11 × 10^10^		
	D2323 2.11 × 10^10^		

^1^ e1 11 e1 22 e1 33 e1 12 e2 12 e2 13 e1 23 e2 11 e2 22 e2 33 e3 12 e3 13 e3 23 are zero.

**Table 3:** Dimensional parameters of the steel pipe model.

Length (mm)	Outside Diameter (mm)	Wall Thickness (mm)
2000	70	4

**Table 4:** The mechanical property parameters of steel material.

	Density (kg/m^3^)	Young’s Modulus (Pa)	Poisson Ratio
Steel ^1^	7850	21 × 10^10^	0.32

^1^ In the real tube, the type of steel is plain carbon steel for which these mechanical properties were obtained from the production certificate of the pipe factory, and then these mechanical properties were input to the ABAQUS material model.

**Table 5:** Calculation of guided wave propagation velocity in the pipe model.

Propagation Distances (mm)	400	800	1200
Propagation time (ms)	0.745	1.502	2.322
Calculated group velocities (ms^−1^)	5369	5263	5167
Theoretical group velocities ^1^ (ms^−1^)	5361	5361	5361
Relative errors(%) ^2^	0.149%	1.828%	3.619%

^1^ The theoretical group velocity is calculated according to the group velocity dispersion curve of L(0, 2) mode at the frequency of 70 kHz shown in Figure 2a; ^2^ $\mathrm{Relative}\;\mathrm{error}(\%)=\frac{\left|\mathrm{Calculated}\;\mathrm{group}\;\mathrm{velocities}|-|\mathrm{Theoretical}\;\mathrm{group}\;\mathrm{velocities}\right|}{\left|\mathrm{Theoretical}\;\mathrm{group}\;\mathrm{velocities}\right|}$.

**Table 6:** Comparison of the evaluation index.

	Healthy	45° Damage	90° Damage
Simulation	0	26.44	34.65
Experiment	0	20.54	30.70
Absolute error ^1^	0	5.9	3.95

^1^ Absolute error=|Simulation| – |Experimental value|.

**Table 7:** Comparison of signal package amplitudes (Volts).

	Healthy	45° Damage	90° Damage
Simulation (V)	0.344	0.163	0.171
Experiment (V)	0.267	0.107	0.148
Absolute error (V)^1^	0.077	0.056	0.023

^1^ Absolute error(V)=|Simulation| – |Experimental value|.

**Table 8:** Comparison of the damage localization (mm).

	Healthy ^1^	45° Damage	90° Damage
Simulation	2032.9	960.5	939
Experiment	2062.6	980.5	971.2
Relative error (%) ^2^	1.44	2.03	3.32

^1^ For the healthy level, the evaluated distance is from the sensor to one of the boundaries; ^2^ $\mathrm{Relative}\;\mathrm{error}(\%)=\frac{\left|\mathrm{Simulation}|-|\mathrm{Experimental}\;\mathrm{value}\right|}{\left|\mathrm{Experimental}\;\mathrm{value}\right|}$.
